# An infant with testicular Fetus-in-fetu in the abdominal cavity: rare case report

**DOI:** 10.3389/fped.2024.1442034

**Published:** 2024-08-12

**Authors:** Mingshuang Luo, Geng Li, Heyun Gao, Wen Zhang

**Affiliations:** Department of Pediatric Surgery, Zhongnan Hospital of Wuhan University, Wuhan, China

**Keywords:** testicular, Fetus-in-fetu, teratoma, clinical manifestation, abdominal cavity

## Abstract

**Background:**

Fetus-in-fetu (FIF) represents an exceedingly rare disease, characterized as an encapsulated and pedunculated vertebrate neoplasm, typically lacking cerebral tissue. The prevalence shows no gender preference. Notably, FIF can cause compressive damage to adjacent organs and tissues, potentially impeding the host's development and maturation.

**Case presentation:**

A four-month-old male infant was identified, during pregnancy, to have a left-sided pelvic mass on ultrasound. Subsequent evaluations suggested the mass could be a FIF, exhibiting active movement. Surgical exploration revealed that the mass's left boundary was connected to the left spermatic cord and vas deferens. Pathological analysis post-surgery showed the absence of testicular tissue, but the presence of skin tissue, cartilage-like structures, and gastrointestinal elements. Additionally, localized tissue resembling vertebrae confirmed the diagnosis of testicular FIF.

**Conclusion:**

An intraperitoneal testicular FIF is extremely rare, with its cause still unknown. This groundbreaking report details the diagnosis and management of such a case. Following a FIF diagnosis, prompt surgical removal is crucial, along with regular follow-up using ultrasound and tumor markers.

## Introduction

A Fetus-in-Fetu (FIF) is defined as an embryonic structure possessing a spinal column and internal organs arranged along this axis, enclosed within a normally developing fetus (1, 2). The occurrence of FIF is extremely rare, with studies suggesting an overall prevalence of approximately 1 in 500,000 (3). About 80% cases of FIF have been found in the retroperitoneum, with additional instances identified in the cranial cavity, oropharynx, neck, mediastinum, spine, and scrotum (4). The diagnosis of FIF relies on radiological examination. Typically, FIF diagnosis culminates in surgical removal as the primary treatment approach, with a generally favorable prognosis (5). However, this study represents the first documented case of intraperitoneal testicular FIF in an infant.

## Case data

A male infant at four months of age presented with a mass in the left pelvic cavity detected during maternal prenatal screening at 29 weeks of gestation. The mother had no history of past miscarriages. There were no familial occurrences of twins or other abnormalities. The child was born naturally in our hospital and showed no postnatal vomiting or other gastrointestinal problems. Surgical intervention was advised by our department upon consultation, however, due to familial reasons, the guardian declined the surgery. Subsequent color Doppler ultrasonography revealed progressive enlargement of the mass, with persistent absence of the left testis and the mass shifting from the left to the right. Urgent surgical intervention was recommended. After deliberation, the guardian agreed to the surgery, but the child developed fever and respiratory tract infection symptoms. The child was hospitalized upon recovery, where a physical examination revealed a mass approximately 4.0 × 3.0 cm in the right lower abdomen with clear boundaries, good mobility, and normal bowel sounds. No palpable testicular tissue was identified in the left scrotum or inguinal area, meanwhile the right testis was palpable in the right scrotum. The child's serum levels of *α*-fetoprotein (AFP), carcinoembryonic antige (CEA), and *β*-human chorionic gonadotropin (*β*-HCG) were within normal ranges.

**Figure 1:**
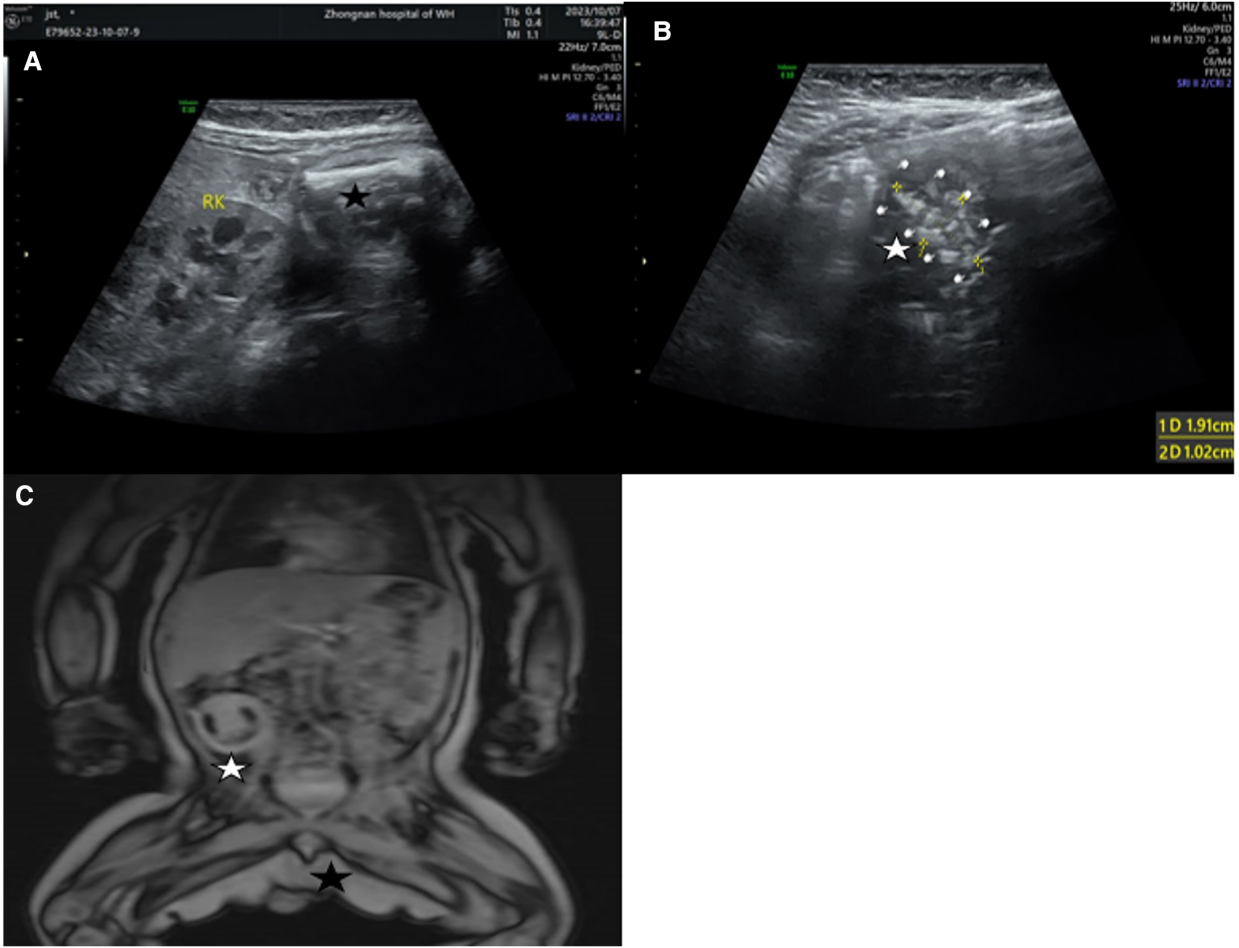
(**A**) Hyperechoic mass in the lower right abdomen and a long bone (black pentagram). (**B**) Irregular spinal hyperechoic area (White pentagram). (**C**) a cystic-solid mass in the right abdomen (White pentagram), left scrotal emptiness (Black pentagram).

Abdominal ultrasound revealed a slightly hyperechoic mass measuring approximately 4.30 × 3.08 × 3.30 cm in the lower right abdomen, with an elongated bone fragment measuring around 2.48 cm (Figure 1A). Additionally, an irregular strong spinal echo, measuring approximately 1.91 × 1.02 cm, raised suspicions of fetal intra-abdominal calcifications (Figure 1B). Magnetic resonance imaging (MRI) of the abdominal and pelvic regions showed a cystic-solid mass with distinct boundaries measuring around 3.60 × 3.10 × 4.40 cm, containing fatty tissue. The left scrotum appeared empty (Figure 1C).

**Figure 2:**
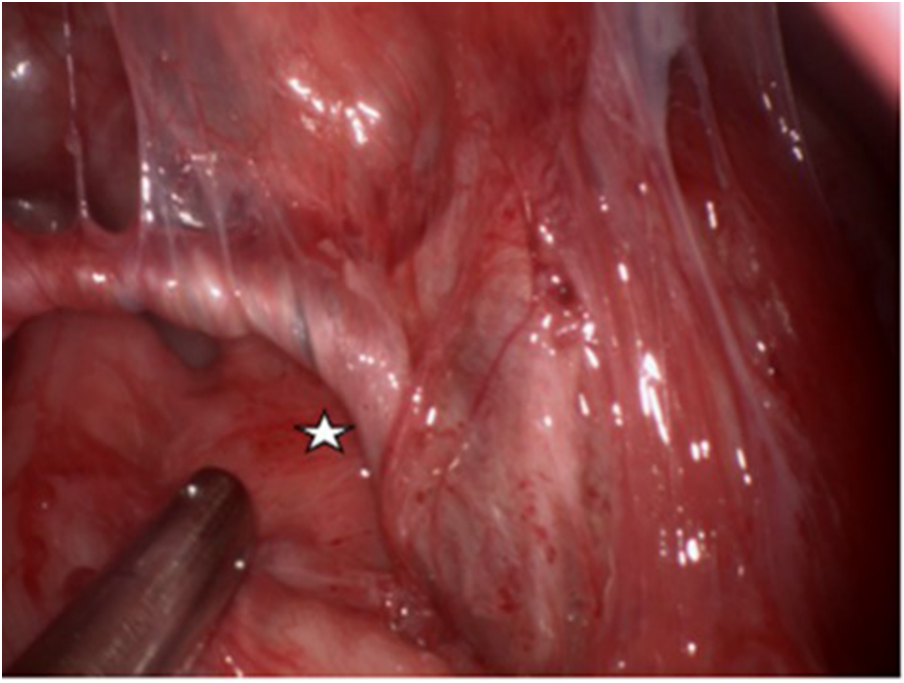
The left wall of the tumor is connected with the left spermatic cord and vas deferens, and the spermatic cord and vas deferens are twisted. (White pentagram).

Following completion of the preoperative evaluation, surgical resection of the suspected FIF was performed under general anesthesia. Intraoperatively, the tumor was found to be connected with the left spermatic cord and vas deferens, with observed torsion (Figure 2). No obvious testicular tissue was identified upon attempting tumor removal. Finally, the resection of the left testis and spermatic cord tissue was performed, while preserving the right testis and spermatic cord tissue. Postoperative pathological assessment confirmed the diagnosis of testicular FIF.

**Figure 3:**
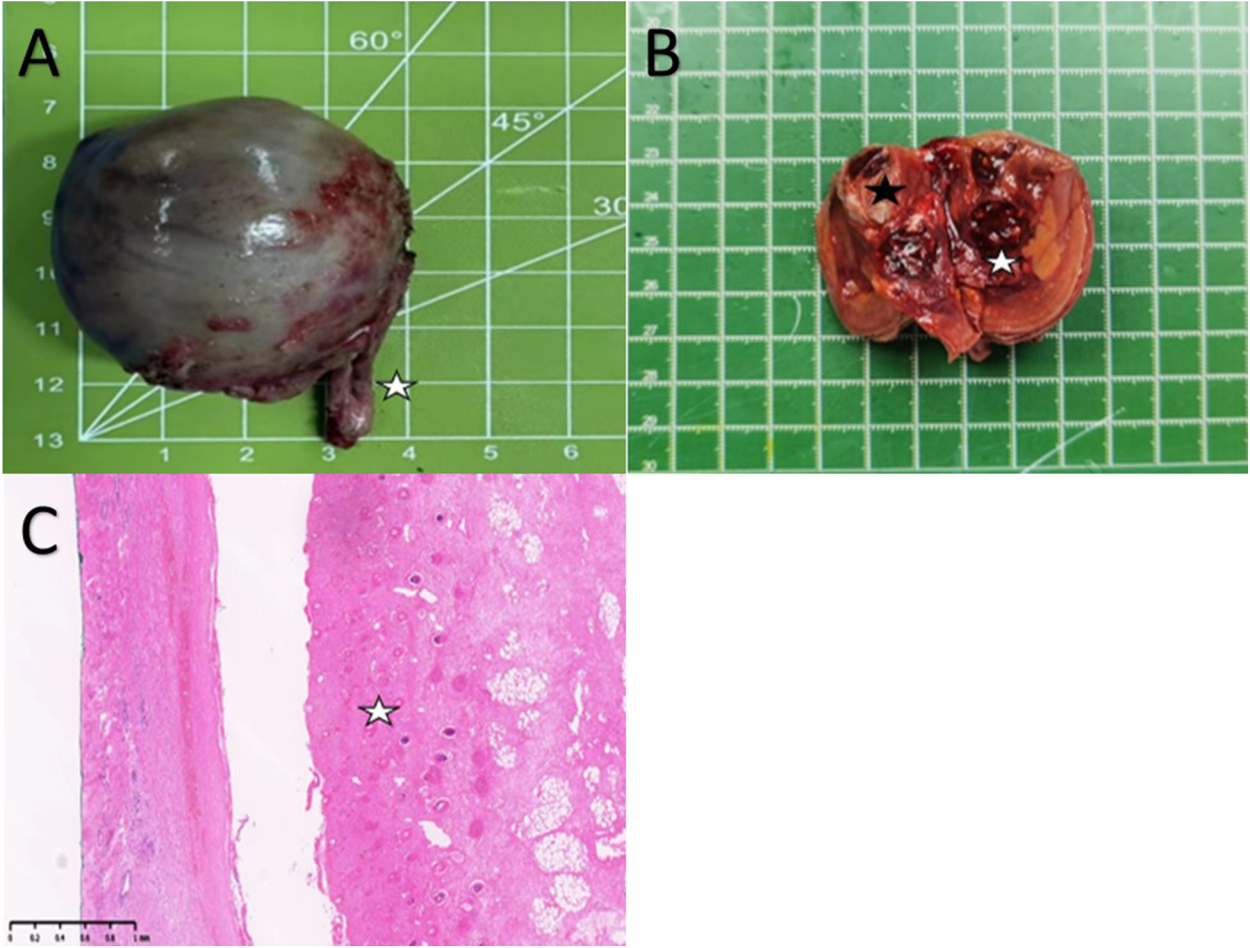
(**A**) A Spermatic cord tissue (white pentagram). (**B**) Cartilage-like tissue (Black pentagram) and local spinal osseous tissue (White pentagram). (**C**) Cartilage hyperplasia (White pentagram).

The postoperative histopathological findings revealed a 4.5 × 3.5 × 3.5 cm gray-brown specimen resembling intact testicular tissue with sections of spermatic cord tissue measuring about 2.5 cm (Figure 3A). After dissection of the specimen, taupe and grayish-yellow tissues were observed, along with identifiable cartilage and long bone tissues. Furthermore, the presence of the spinal structure was also confirmed (Figure 3B). Microscopic examination with HE staining displayed irregular bone and cartilage proliferation (Figure 3C). Microscopic analysis showed presence of cartilage, intact bone cortex, and bone marrow cavity within the long bone. Although skin layers (epidermis, dermis), subcutaneous tissue, and digestive tract structures were observable, clear testicular parenchymal structures such as seminiferous tubules were absent.

Following the surgery, the child had a smooth recovery and was discharged on the 6th postoperative day. During the 6-month follow-up period, no postoperative complications were reported.

## Discussion

FIF is an extremely uncommon condition, characterized by a unique type of conjoined twins in monozygotic twins, which can be classified into endogenous FIF and exogenous FIF. Endogenous FIF is seen more commonly than exogenous fetus (6). FIF cases are more prevalent in singleton pregnancies, few in multiple pregnancies, and are typically identified in infants and young children, with very few cases occurring in adulthood (7).

The prevailing view among scholars is that FIF stems from the degeneration of twins or teratomas. The mainstream view is the monozygotic twin theory, proposing that unequal division of the fertilized egg at the early embryonic stage, resulting in the smaller inner cell mass being enveloped by the larger cell mass, which leading to the differentiation and development of the fetus. This theory can be substantiated by determining if the FIF individual and the host share the same gender, blood type, and DNA alleles (8). However, the teratoma degeneration theory suggests that teratomas could transform into highly differentiated FIF structures.

The clinical presentations of FIF are primarily associated with the space-occupying effects of masses, leading to symptoms such as vomiting, dyspnea, abdominal distension, jaundice, intestinal obstruction, and compressive damage to the kidney. The predominant clinical feature is the presence of abdominal masses during infancy and early childhood (9).

The diagnosis of FIF heavily relies on imaging studies. Spines and vertebrae are identified in only about 50% of reported cases (10). However, ultrasound is capable of directly visualizing the number of fetuses in the amniotic sac and the site of fusion of conjoined fetuses (11). CT and MRI imaging offer exceptional tissue resolution and advanced image processing capabilities, enabling precise assessment of the anatomy, fusion site, bone structure, blood supply, and vascular anatomy of early conjoined twins, facilitating surgical planning (12, 13). Considering the patient's young age and the potential radiation risks associated with CT scans, MRI was chosen for preoperative evaluation. The diagnosis of FIF necessitates specific characteristics, such as the mass being encapsulated, partially or fully covered by skin, displaying distinct anatomical structures, and being connected to the host through a pedicle containing blood vessels (14).

Distinguishing FIF from teratoma is primarily achieved through pathological examination. Teratomas typically contain hair, teeth, and some intestinal components, but lack fully developed organs like long bones, limbs, and spine that are commonly found in FIF. Compared to the differential diagnosis of teratoma, meconium pseudocyst can be differentiated through imaging studies, showcasing abdominal calcification and predominantly ascites, whereas calcification in FIF occurs in the spine and long bones, with the absence of ascites (15).

Once FIF is diagnosed, prompt surgical intervention is essential. Whether opting for traditional laparotomy or minimally invasive surgery, it is crucial to adequately expose the surgical field, identify the supplying vessels, and approach the FIF as a neoplastic entity to ensure complete excision of both the FIF and its capsule. Despite FIF being characterized as a benign condition, there exists a certain risk of malignant transformation. A documented case of malignant FIF has been reported (16). Hence, prolonged post-operative monitoring and regular reassessment are imperative for children with FIF.

## Conclusion

Intraperitoneal testicular FIF is a highly uncommon condition, and the underlying causes are not yet fully understood. This research is the initial attempt to extensively outline the diagnosis and management of intraperitoneal testicular FIF. Upon FIF diagnosis, prompt surgical removal is imperative, followed by routine post-operative monitoring through color ultrasound and tumor marker assessments.

## Data Availability

The original contributions presented in the study are included in the article/Supplementary material, further inquiries can be directed to the corresponding author.
